# Associations of maternal exposure to fine particulate matter with preterm and early-term birth in high-risk pregnant women

**DOI:** 10.1186/s41021-022-00239-0

**Published:** 2022-03-15

**Authors:** Kaixin Cao, Hongyan Jin, Haoxin Li, Mengmeng Tang, Jianhong Ge, Zekang Li, Xiaoyun Wang, Xuetao Wei

**Affiliations:** 1 School of Public Health, Peking University, 100191 Beijing, China; 2 Beijing Key Laboratory of Toxicological Research and Risk Assessment for Food Safety, Peking University, 100191 Beijing, China; 3 Peking University First Hospital, 100191 Beijing, China

**Keywords:** PM_2.5_, High-risk pregnant women, Preterm birth, Early-term birth

## Abstract

**Background:**

Environmental pollution is a risk factor for adverse birth outcomes, especially preterm birth (PTB) and early-term birth (ETB). It has been revealed that exposure to fine particulate matter (PM_2.5_) during pregnancy increase the prevalence of PTB. However, the relationship between PM_2.5_ exposure and ETB has not been elucidated. In high-risk pregnancies, whether PM_2.5_ exposure will bring higher risk of PTB and ETB than in normal pregnancies is still unclear, and the susceptible exposure window is obscure. Therefore, it is worthy of assessing the risk on PTB and ETB and identifying the susceptible exposure windows of PM_2.5_ exposure in high-risk pregnant women.

**Results:**

This paper collected the clinical data of 7974 singletons, high-risk pregnant women in Peking University First Hospital from 2014 to 2018, and analyzed them using logistic regression and stratified analysis. We observed that exposure to high-level (≥ 75 µg/m^3^) of PM_2.5_ during the third trimester of pregnancy increases the risk of PTB and ETB (PTB: odds ratio[OR] = 1.43, 95% confidence interval [CI]:1.05–1.93. ETB: OR = 1.29, 95%CI: 1.09–1.54). Furthermore, the effects of each 10ug/m^3^ increase in PM_2.5_ on PTB and ETB were significant during the third trimester (PTB: OR = 1.35, 95%CI:1.16–1.58. ETB: OR = 1.12, 95%CI:1.02–1.22) and the entire pregnancy (PTB: OR = 6.12, 95%CI:4.27–8.89. ETB: OR = 1.96, 95%CI:1.59–2.43) in the high-level exposure group.

**Conclusions:**

These results suggest that high-level PM_2.5_ exposure during pregnancy is associated with high risk of PTB and ETB in high-risk pregnancies. The third trimester of pregnancy is speculated to be the susceptible exposure window.

**Supplementary Information:**

The online version contains supplementary material available at 10.1186/s41021-022-00239-0.

## Introduction

Preterm birth (PTB), defined as babies born before 37 completed weeks of pregnancy, has become an increasing global health problem [1, 2]. The incidence of PTB is increasing globally, ranging from 7.4 to 13.5% in different regions [3, 4]. Preterm infants are at high risk of death and disability [5]. As the leading cause of death in children under five years of age, PTB can lead to several complications such as dyspnea, neurodevelopmental sequelae and intracranial hemorrhage [6, 7]. Moreover, PTB is the ninth leading cause of disability-adjusted life-years globally [8]. Contrary to the past belief that neonatal outcomes for term births (37–40 weeks’ gestation) were uniform and good, early-term birth (ETB, 37–38 weeks’ gestation) was recently found to have poorer neonatal outcomes, especially respiratory morbidity, and long-term health outcomes such as educational outcomes, than full-term birth (39–40 weeks’ gestation) [9–15].

PTB and ETB are multi-factorial processes, and the causation of spontaneous preterm delivery remains unidentified in up to half of all cases [16, 17]. The WHO reported that environmental factors represent 6% of the causation of adverse pregnancy outcomes [18]. Due to the updated satellite and monitoring data, air pollutants, especially PM_2.5_, have drawn much more attention in recent years. Current studies have not reached a consensus on the relationship between PM_2.5_ exposure and PTB. Some epidemiological studies observed a significant positive association between PM_2.5_ exposure and PTB in different areas where the average PM_2.5_ concentration range from 10 to 70 µg/m^3^ [19–29], however, others do not [30–32]. Moreover, only one research conducted in China has explored the association between PM_2.5_ and ETB (hazard ratio = 1.09 for each 10 µg/m^3^ increase in PM_2.5_ over the entire pregnancy, 95%CI: 1.09–1.10) [28].

High-risk pregnant women refer to those who are prone to high blood pressure, diabetes, fetal malformations, miscarriage, premature delivery and other risks during pregnancy. Exposure to PM2.5 in high-risk pregnant women may promote preterm birth and have a greater impact on adverse pregnancy outcomes through interaction with risk factors compared with healthy mothers. A growing body of studies has explored the association between maternal exposure to PM_2.5_ and PTB in China [33–35]. However, these researches were conducted in a relatively healthy population and seldom adjusted potential confounders like maternal medical conditions.

Our study was designed to focus on the high-risk pregnant women in Beijing during 2014–2018. Meanwhile, the detailed high-risk factors of each subject were collected. The effects of PM_2.5_ exposure on PTB and ETB were evaluated, and sensitive periods of PM_2.5_ exposure were explored.

## Methods

### Study population

The study population for this study was the mothers have been diagnosed as high-risk individuals during pregnancy according to *Beijing Risk Assessment Form of Pregnancy* in Peking University, First Hospital. Based on the Hospital’s maternal high-risk database, 9250 women who conceived and delivered between Jan 1st, 2014 to Dec 31st, 2018 were eligible for inclusion. The main exclusion criteria included multiple-gestation pregnancies, stillbirth and key information missing (e.g., date of delivery, gestational age and home address). After exclusion, the cohort finally includes 7974 singleton live birth pregnancies for further analysis. The details are given in Fig. S1 (see Supplementary information).

Data for the current study were obtained from Peking University, First Hospital, including birth records and maternal high-risk database. Specifically, birth records registered by obstetric nurse contains the information of pregnancy outcome. The maternal high-risk database is specifically for high-risk pregnant women tracking the occurrence of risk factors such as alcohol consumption, exposure to smoking, and most importantly, the underlying maternal high-risk medical conditions throughout pregnancy. Moreover, the detailed home address of pregnant women is recorded in the high-risk database, which is the basis for our exposure assessment.

### Exposure window and exposure assessment

To explore the susceptible window of PM_2.5_ exposure during pregnancy, we defined four exposure periods: the entire pregnancy, the first trimester (1–13 weeks), the second trimester (14–26 weeks), and the third trimester (27 weeks-birth).

The data on PM_2.5_ exposure for each individual from pregnancy to childbirth was obtained from Beijing Municipal Environmental Monitoring Center and calculated using inverse distance weighted interpolation, which has been demonstrated to be the best approach for our study [36]. Briefly, hourly concentrations of PM_2.5_, recorded by 35 monitoring stations across the city of Beijing from 2014 to 2018, were collected and then they were converted into daily averages. Using inverse distance weighted interpolation, we estimated the daily mean level of PM_2.5_ exposure for each pregnant woman based on their home address and pregnancy time. The geographical distribution map of the participants’ home addresses and nearby monitoring sites are shown in Fig. S2 (see Supplementary information).

For exploring the sensitive exposure window, the daily average concentrations of PM_2.5_ in four exposure periods—the entire pregnancy, first trimester, second trimester, and third trimester were calculated using daily mean level above and were categorized as high-level exposure if the daily average concentration over the specified time period was greater than 75 µg/m^3^, while low-level exposure with PM_2.5_ less than 75 µg/m^3^, taking account of the Chinese ambient air quality standard for 24-hour average of PM_2.5_ [37].

### Outcome and covariates

Our main outcomes were preterm birth and early term birth. PTB was defined as delivery prior to 37 completed weeks of gestational age and ETB was defined as delivery from 37 to 38 weeks of gestational age.

The selected covariates contain maternal age (< 35 or ≥ 35 years of age), parity (1, 2 or ≥ 3), infant sex (male or female), season of conception (spring: March to May, summer: June to August, autumn: September to December, winter: November to February), year of conception, pregnancy body mass index (BMI in kg/m^2^, < 24 or ≥ 24), hazardous poison exposure (yes or no), mode of delivery( cesarean section or vaginal delivery) and the underlying maternal high-risk medical conditions: hyperglycemia (yes or no), hypertension (yes or no), scarred uterus (yes or no), uterine fibroids (yes or no), ovarian cyst (yes or no) and in vitro fertilization (yes or no). Hazardous poison exposure was defined as exposure to smoking, drinking, occupational poison/contraindication, or radiation during pregnancy.

### Statistical analysis

We used χ^2^ test to compare the difference among pregnant outcomes. The associations between pregnant outcomes and PM_2.5_ exposure were estimated using logistic regression analysis, and the results were reported as ORs (odds ratio) with their 95%CIs (confidence interval). In the primary analysis, ORs for high-level PM_2.5_ exposure during the first, second and third trimester as well as over the entire pregnancy for each outcome (ETB and PTB) were estimated from separate models. In the secondary analysis, PM_2.5_ was modeled as a continuous variable, and the relationships between PM_2.5_ exposure increased per 10 µg/m^3^ and the risk of each outcome were explored through stratified analyses in high-level exposure group and low-level exposure group respectively. The effects of maternal age, BMI, hazardous poison exposure, parity, infant sex, season of conception, the year of conception, mode of delivery and the underlying maternal high-risk medical factors were adjusted. In addition, the level of PM_2.5_ exposure (high-level or low-level) during earlier stages of pregnancy was also adjusted in the later stage of pregnancy models.

Sensitivity analyses were performed to examine the robustness of results. Specifically, we repeated the primary analysis at non-hyperglycemia and non-hypertension populations, and we also did stratified analyses by the mode of delivery. All analyses were performed using R version 3.6.0. Comparison with a two-sided probability value < 0.05 was considered statistically significant.

## Results

A total of 7974 high-risk pregnant women with live singletons birth were included. The incidence rate of PTB was 8.18% (652/7974) and ETB was 33.94% (2706/7974). Women of advanced maternal age (≥ 35 years of age) accounted for 49.02% of the study population. Half of the mothers (49.71%) reported this birth as their first child, and half of the mothers (50.69%) delivered by caesarean section. After preliminary statistical analysis, preterm birth rates and early term birth rates were higher among mothers older than 35 years old, delivered by caesarean section, as well as mothers diagnosed with hypertension or hyperglycemia (Table 1, Table S1 see Supplementary information). Table 1 and S1 summarized the detailed characteristics of the study population.

**Table 1:** Characteristics of mothers of preterm and term infants

Characteristics	Total		Preterm birth		Term birth^*^		*P*
	*N*=7974(100%)		*N*=652(8.18%)		*N*=7322(91.82%)		
	n	(%)	n	(%)	n	(%)	
Maternal age ≥ 35(%)	3909	49.02	329	50.46	3580	48.89	0.47
BMI≥24 (%)	408	5.12	40	6.13	368	5.03	0.25
Exposure to hazardous poison (%)	93	1.17	7	1.07	86	1.17	0.97
Number of previous deliveries (%)							0.01
0	3964	49.71	351	53.83	3613	49.34	
1	3908	49.01	288	44.17	3620	49.44	
2	102	1.28	13	1.99	89	1.22	
Number of previous pregnancies (%)							0.13
0	2351	29.48	197	30.21	2154	29.42	
1	2820	35.36	210	32.21	2610	35.65	
2	1637	20.53	132	20.25	1505	20.55	
≥3	1166	14.62	113	17.33	1053	14.38	
Baby’s sex of male (%)	4107	51.50	348	53.37	3759	51.34	0.34
In Vitro Fertilization (%)	763	9.57	72	11.04	691	9.44	0.21
Delivery by cesarean section (%)	4042	50.69	418	64.11	3624	49.49	<0.001
Hyperglycemia (%)	189	2.37	23	3.53	166	2.27	0.06
Hypertension (%)	151	1.89	37	5.67	114	1.56	<0.001
Scarred uterus (%)	2223	27.88	190	29.14	2033	27.77	0.48
Ovarian cyst (%)	186	2.33	16	2.45	170	2.32	0.94
Uterine fibroids (%)	976	12.24	83	12.73	893	12.20	0.74
Season of conception (%)							0.19
Spring	2042	25.61	185	28.37	1857	25.36	
Summer	1873	23.49	135	20.71	1738	23.74	
Autumn	1934	24.25	153	23.47	1781	24.32	
Winter	2125	26.65	179	27.45	1946	26.58	
Year of conception (%)							<0.001
2014	22	0.28	0	0.00	22	0.30	
2015	1557	19.53	110	16.87	1447	19.76	
2016	2723	34.15	225	34.51	2498	34.12	
2017	2932	36.77	228	34.97	2704	36.93	
2018	740	9.28	89	13.65	651	8.89	

*term birth: delivery ≥ 37 weeks of gestation

The average level of PM_2.5_ during the first, second and third trimester and the entire pregnancy was 70.72 µg/m^3^, 69.02 µg/m^3^, 66.15 µg/m^3^ and 68.60 µg/m^3^, respectively, their interquartile range was also showed in Table S2 (see Supplementary information). Table 2 shows crude and adjusted odd ratios and 95% confidence intervals for PTB and ETB in participants exposed to high-level PM_2.5_ during different periods of pregnancy. After adjustment for covariates, high-level PM_2.5_ exposure during the third trimester increased risk of preterm birth and early term birth, the adjusted ORs (95%CI) were 1.43 (95%CI: 1.05–1.93) and 1.29 (95%CI: 1.09–1.54), respectively.

**Table 2:** Crude and adjusted odds ratios and their 95% CI for high-level PM_2.5_ of preterm birth and early term birth

Outcomes	Trimester 1		Trimester 2		Trimester 3		Entire pregnancy	
	OR (95% CI)	*P*	OR (95% CI)	*P*	OR (95% CI)	*P*	OR (95% CI)	*P*
Crude model^1^								
Full-term birth	1.00		1.00		1.00		1.00	
Preterm birth	0.96(0.79,1.15)	0.654	0.90(0.74,1.10)	0.324	1.29(1.04,1.59)	0.021	0.84(0.71,1.00)	0.054
Early term birth	0.83(0.75,0.93)	0.002	1.06(0.94,1.18)	0.331	1.39(1.23,1.58)	< 0.001	1.03(0.93,1.13)	0.611
Adjusted model^2^								
Full-term birth	1.00		1.00		1.00		1.00	
Preterm birth	1.12(0.86,1.47)	0.396	1.00(0.76,1.32)	0.984	1.43(1.05,1.93)	0.021	0.68(0.50,0.94)	0.019
Early term birth	0.90(0.77,1.06)	0.204	0.99(0.84,1.17)	0.923	1.29(1.09,1.54)	0.004	0.87(0.72,1.05)	0.156

High-level PM_2.5_: average concentration over the specified time period ≥ 75 µg/m^3^

1: Logistic regression model, adjusted for maternal age and BMI

2: Logistic regression model, adjusted for maternal age, BMI, exposure to hazardous poison, number of previous deliveries, the season of conception, the year of conception, sex of the baby, mode of delivery, hyperglycemia, hypertension, scarred uterus, uterine fibroids, ovarian cyst, in vitro fertilization and the PM_2.5_ exposure level during earlier stages of pregnancy

Results for the associations of PTB and ETB with 10 µg/m^3^ increase in PM_2.5_ exposure based on exposure level stratification are presented in Table 3. Under high exposure condition (PM_2.5_≥75 µg/m^3^), we observed PM_2.5_ exposure in the third trimester was associated with an increased risk of PTB and ETB (for preterm birth, OR = 1.35, 95%CI: 1.16–1.58; and for early term birth, OR = 1.12, 95%CI: 1.02–1.22). Similarly, the effects of PM_2.5_ exposure on PTB and ETB were significant during the entire pregnancy (for preterm birth, OR = 6.12, 95%CI: 4.27–8.89; and for early term birth, OR = 1.96, 95%CI: 1.59–2.43) among high-level exposure group (PM_2.5_≥75 µg/m^3^). However, no significant associations between PM_2.5_ exposure and PTB or ETB were observed at low exposure condition.

**Table 3:** Adjusted odds ratios and 95%CIs of preterm birth and early term birth for each 10 µg/m^3^ increment in PM_2.5_ exposure during trimesters and the entire pregnancy

	High-level PM_2.5_					Low-level PM_2.5_				
	Full term birth	Preterm birth		Early term birth		Full term birth	Preterm birth		Early term birth	
		OR (95% CI)	*P*	OR (95% CI)	*P*		OR (95% CI)	*P*	OR (95% CI)	*P*
Trimester 1	1.00	0.91(0.80,1.04)	0.161	0.97(0.89,1.05)	0.444	1.00	0.95(0.82,1.10)	0.503	0.95(0.87,1.03)	0.210
Trimester 2	1.00	0.89(0.77,1.03)	0.122	0.99(0.90,1.07)	0.741	1.00	0.86(0.74,1.01)	0.071	1.02(0.93,1.12)	0.705
Trimester 3	1.00	1.35(1.16,1.58)	< 0.001	1.12(1.02,1.22)	0.021	1.00	0.93(0.81,1.06)	0.249	0.97(0.89,1.05)	0.431
Entire pregnancy	1.00	6.12(4.27,8.89)	< 0.001	1.96(1.59,2.43)	< 0.001	1.00	1.01(0.79,1.30)	0.917	0.99(0.85,1.14)	0.850

High-level PM_2.5_: average concentration over the specified time period ≥ 75 µg/m^3^

Low-level PM_2.5_: average concentration over the specified time period < 75 µg/m^3^

Logistic regression model, adjusted for maternal age, BMI, exposure to hazardous poison, number of previous deliveries, the season of conception, the year of conception, sex of the baby, mode of delivery, hyperglycemia, hypertension, scarred uterus, uterine fibroids, ovarian cyst, in vitro fertilization and the PM_2.5_ exposure level during earlier stages of pregnancy

**Fig. 1:**
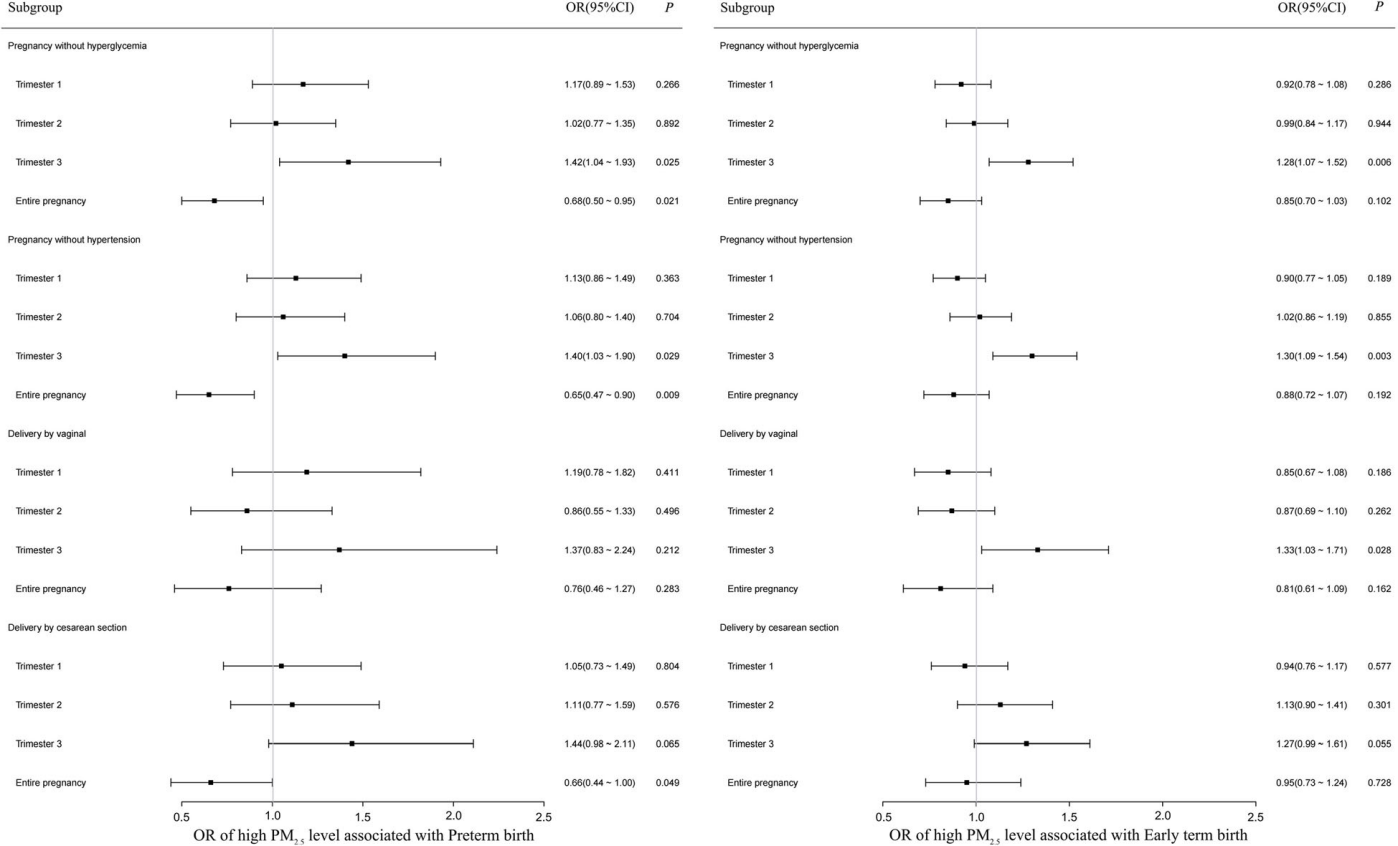
Sensitivity analysis of high-level PM_2.5_ exposure associated with preterm birth and early term birth in each subgroup population. Logistic regression model, adjusted for maternal age, BMI, exposure to hazardous poison, number of previous deliveries, the season of conception, the year of conception, sex of the baby, mode of delivery, hyperglycemia, hypertension, scarred uterus, uterine fibroids, ovarian cyst, in vitro fertilization and the PM_2.5_ exposure level during earlier stages of pregnancy

To evaluate the robustness of the results, we conducted sensitivity analyses, the results are shown in Fig. 1. For early term birth, the sensitivity analyses among subgroup of non-hypertension and non-hyperglycemia as well as among vaginal delivery individuals did not substantially change the results. However, in the subgroup analysis of cesarean section, compared with the results of the whole population, the effect of high-level PM_2.5_ during the third trimester was attenuated, and the difference was no statistically significant. The results of sensitivity analyses for PTB were similar to ETB.

## Discussion

We evaluated the associations between exposure to PM_2.5_ and PTB as well as ETB in high-risk pregnant women. The result indicated that exposure to PM_2.5_ during the third trimester or throughout pregnancy was positively associated with PTB and ETB.

At stratified analysis, we found a close association between PM_2.5_ exposure during the entire pregnancy and PTB on the high exposure condition. It is consistent with recent research. Studies including meta-analysis, two national birth cohort studies and investigations of individual cities in China all drew similar conclusions, indicating an increased risk of PTB induced by PM_2.5_ exposure [20, 27, 28, 33, 38].

As for the susceptible window of PM_2.5_ exposure during pregnancy, there is no consistent conclusions. In our study, we observed PM_2.5_ exposure in the third trimester was associated with an increased risk of PTB and ETB. A retrospective cohort study in China found that the correlation between PM_2.5_ exposure and increased risk of PTB was most pronounced in the third trimester (HR = 1.06, 95%CI:1.06–1.07 for each 10 µg/m^3^ increase in PM_2.5_) [27]. Meanwhile, studies in Shanghai, China (OR = 1.06, 95%CI:1.01–1.12 for each 10 µg/m^3^ increase in PM_2.5_) [33], as well as in Guangzhou, China [35] also found PM_2.5_ exposure in the third trimester was strongly responsible for the increased cases of PTB. However, two recent meta-analysis researches combining previous studies found no association of PTB with PM_2.5_ exposure during the third trimester, and the ORs(95%CI) were 1.02(0.99 ~ 1.04) and 1.08(0.99 ~ 1.17), respectively [20, 38]. Regarding early term birth, only one study in China has investigated the associations between PM_2.5_ and ETB, reporting a significant association between PM_2.5_ and ETB at specific times in three trimesters and throughout pregnancy [28].

Compared with previous studies, this study shows stronger detrimental associations between PM_2.5_ exposure with PTB and ETB. The exposure level, study design, and sample population may potentially contribute to the difference. Firstly, the population in this study was exposed to a much higher level of PM_2.5_ than studies conducted in other regions (e.g., Europe and USA) [31, 32, 39]. Secondly, we used term delivery (delivery from 39 to 40 weeks) as a control group in contrast to previous studies that used term delivery (≥ 37 full weeks) as a control. This study, and related studies have shown that PM_2.5_ can induce an elevated risk of ETB [28]. Therefore, changes in the selected control group may have resulted in higher outcomes compared to existing studies based on the full-term birth control group. Furthermore, we focused on the high-risk pregnant women. Blencowe, et al. [4] has reported diseases, such as diabetes, hypertension, are risk factors for PTB. Moreover, relative to the healthy pregnant population, women with pre-pregnancy diabetes, asthma or preeclampsia were more sensitive to PM_2.5_ [26]. As for the other individual risk factors like age and parity, the proportions of pregnant women in our study who were over 35 years old and had previous pregnancies were up to 49.02% and 70.52% respectively, which are much higher than those in prior studies [27, 40]. Finally, variations in the source and composition of PM_2.5_ may also be one of the reasons for the different results, as it has been reported that several sources of PM_2.5_ and specific PM_2.5_ components are associated with adverse pregnancy outcomes [41–44].

Some merits of this study. Firstly, we collected the maternal high-risk medical conditions during pregnancy of each individual, and adjusted these potential confounders in our statistical analysis, given their documented association with PTB [45]. Secondly, PM_2.5_ exposure in this study was predicated using inverse distance weight based on ground-monitoring data. In our previous methodological studies, this interpolation method showed higher prediction accuracy with a root mean squared error of 17.97 µg/m^3^ [36], which may be mainly due to the high density of monitoring stations. Finally, we try to explore the association between PM_2.5_ and ETB. As far as we know, there have been growing studies focused on the association between air pollution and PTB. However, a few researches reported the impacts of air pollution on ETB. Previous research in obstetrics and gynecology indicated those neonatal outcomes varied depending on the timing of delivery within the period for 3 weeks before until 2 weeks after the estimated date of delivery [9, 46]. Base on the available evidence, we selected the subgroup of full-term birth (39–40 weeks of gestation) as our control group and identified the harmful effect of maternal PM_2.5_ exposure on ETB. The result would extend our understanding of the impact of PM_2.5_ exposure on pregnant outcome.

Our research also has limitations. Firstly, quantification of an individual’s exposure is imprecise since personal sampling equipment is not practical for population cohort studies. Secondly, other air pollutants and their ambient concentrations are not considered. Synergistic effects between PM_2.5_ and other air pollution have been reported in PTB [47]. Finally, despite the statistical adjustment for medical conditions, the other personal factors like education level, household income, mental state, and work pressure are not considered due to unavailability of this information. A previous study reported that adverse health effects due to mental health may be amplified during pregnancy, and increased the risk of adverse pregnancy outcomes such as preterm birth [48].

## Conclusions

Taken together, the results of this study suggest that exposure to high-level PM_2.5_ during the third trimester of pregnancy can increase the risk of preterm birth and early term birth in high-risk pregnant women. The findings from our study indicate that the third trimester of pregnancy might be the sensitive exposure window. Further, research with a larger sample size in the high-risk pregnant population is needed to determine the modified effect of high-risk factors in developing appropriate health care.

## Supplementary information

**Additional file 1.**

## Data Availability

Not applicable.

## Abbreviations

PTB: Preterm birth
ETB: Early term birth
PM_2.5_: Fine particulate matter
OR: Odds ratio
CI: Confidence interval
