# Supplementary material for: Associations of maternal exposure to fine particulate matter with preterm and early-term birth in high-risk pregnant women
Source: Genes Environ. 2022 Mar 15;44:9. doi: 10.1186/s41021-022-00239-0 (PMC8922917; doi:10.1186/s41021-022-00239-0)
Supplement: Supplementary file 1 — Additional file 1. [file 41021_2022_239_MOESM1_ESM.docx]

**Supplementary Materials**

**Associations of maternal exposure to fine particulate matter with preterm and early-term birth in high-risk pregnant women**

Kaixin Cao^a b c1^, Hongyan Jin^c1^, Haoxin Li^a^, Mengmeng Tang^a b^, Jianhong Ge^a b^, Zekang Li^a b^, Xiaoyun Wang^a b^, Xuetao Wei^a, b*^

^a^ School of Public Health, Peking University, Beijing China, 100191.

^b^ Beijing Key Laboratory of Toxicological Research and Risk Assessment for Food Safety, Peking University, Beijing China, 100191. Electronic address: weixt@bjmu.edu.cn

^c^ Peking University, First Hospital, Beijing China, 100191. Electronic address: maggijhy@163.com

*Correspondence: Tel: +86 10 82801520 Fax: +86 10 82801520

E-mail addresses: [weixt@bjmu.edu.cn](mailto:weixt@bjmu.edu.cn)

^1^Kaixin Cao and Hongyan Jin have contributed equally to this work.

**Table of Contents:**

Table S1. Characteristics of preterm, early term and full-term infants.

Table S2. Summary of estimated PM_2.5_ exposure of the four exposure periods (μg/m^3^).

Fig. S1. Flow chart of study population.

Fig. S2. The geographical distribution map of the research objects’ home addresses and monitoring sites in Beijing.

Table S1. Characteristics of preterm, early term and full-term infants

| Characteristics | Preterm birth(N=652) | | Early term birth(N=2706) | | Full-term birth(N=4220) | | *P* |
| --- | --- | --- | --- | --- | --- | --- | --- |
|  | n | （%） | n | （%） | n | （%） |  |
| Maternal age ≥ 35(%) | 329 | 50.46 | 1417 | 52.37 | 2024 | 47.96 | <0.01 |
| BMI≥24 (%) | 40 | 6.13 | 157 | 5.80 | 189 | 4.48 | 0.02 |
| Exposure to hazardous poison (%) | 7 | 1.07 | 25 | 0.92 | 53 | 1.26 | 0.44 |
| Number of previous deliveries (%) |  |  |  |  |  |  | <0.001 |
| 0 | 351 | 53.83 | 1090 | 40.28 | 2243 | 53.15 |  |
| 1 | 288 | 44.17 | 1559 | 57.61 | 1947 | 46.14 |  |
| 2 | 13 | 1.99 | 57 | 2.11 | 30 | 0.71 |  |
| Number of previous pregnancies (%) |  |  |  |  |  |  | <0.001 |
| 0 | 197 | 30.21 | 653 | 24.13 | 1317 | 31.21 |  |
| 1 | 210 | 32.21 | 997 | 36.84 | 1506 | 35.69 |  |
| 2 | 132 | 20.25 | 613 | 22.65 | 829 | 19.64 |  |
| ≥3 | 113 | 17.33 | 443 | 16.37 | 568 | 13.46 |  |
| Baby’s sex of male (%) | 348 | 53.37 | 1435 | 53.03 | 2149 | 50.92 | 0.17 |
| In Vitro Fertilization (%) | 72 | 11.04 | 234 | 8.65 | 419 | 9.93 | 0.09 |
| Delivery by cesarean section (%) | 418 | 64.11 | 1599 | 59.09 | 1907 | 45.19 | <0.001 |
| Hyperglycemia (%) | 23 | 3.53 | 89 | 3.29 | 74 | 1.75 | <0.001 |
| Hypertension (%) | 37 | 5.67 | 80 | 2.96 | 33 | 0.78 | <0.001 |
| Scarred uterus (%) | 190 | 29.14 | 1017 | 37.58 | 998 | 23.65 | <0.001 |
| Ovarian cyst (%) | 16 | 2.45 | 54 | 2.00 | 94 | 2.23 | 0.70 |
| Uterine fibroids (%) | 83 | 12.73 | 303 | 11.20 | 530 | 12.56 | 0.21 |
| Season of conception (%) |  |  |  |  |  |  | <0.001 |
| Spring | 185 | 28.37 | 778 | 28.75 | 1007 | 23.86 |  |
| Summer | 135 | 20.71 | 664 | 24.54 | 971 | 23.01 |  |
| Autumn | 153 | 23.47 | 580 | 21.43 | 1107 | 26.23 |  |
| Winter | 179 | 27.45 | 684 | 25.28 | 1135 | 26.90 |  |
| Year of conception (%) |  |  |  |  |  |  | <0.01 |
| 2014 | 0 | 0.00 | 5 | 0.18 | 17 | 0.40 |  |
| 2015 | 110 | 16.87 | 544 | 20.10 | 823 | 19.50 |  |
| 2016 | 225 | 34.51 | 948 | 35.03 | 1428 | 33.84 |  |
| 2017 | 228 | 34.97 | 967 | 35.74 | 1587 | 37.61 |  |
| 2018 | 89 | 13.65 | 242 | 8.94 | 365 | 8.65 |  |

Table S2. Summary of estimated PM_2.5_ exposure of the four exposure periods (μg/m^3^)

|  | N | P_5_ | P_25_ | P_50_ | P_75_ | P_95_ | Mean | Standard deviation |
| --- | --- | --- | --- | --- | --- | --- | --- | --- |
| Entire | 7974 | 50.36 | 55.17 | 67.40 | 83.37 | 88.91 | 68.60 | 14.32 |
| Trimester 1 | 7974 | 46.45 | 55.34 | 64.72 | 75.39 | 119.85 | 70.72 | 21.88 |
| Trimester 2 | 7974 | 43.62 | 52.86 | 62.98 | 74.76 | 121.08 | 69.02 | 23.02 |
| Trimester 3 | 7974 | 40.15 | 51.10 | 60.11 | 72.06 | 119.84 | 66.15 | 23.04 |


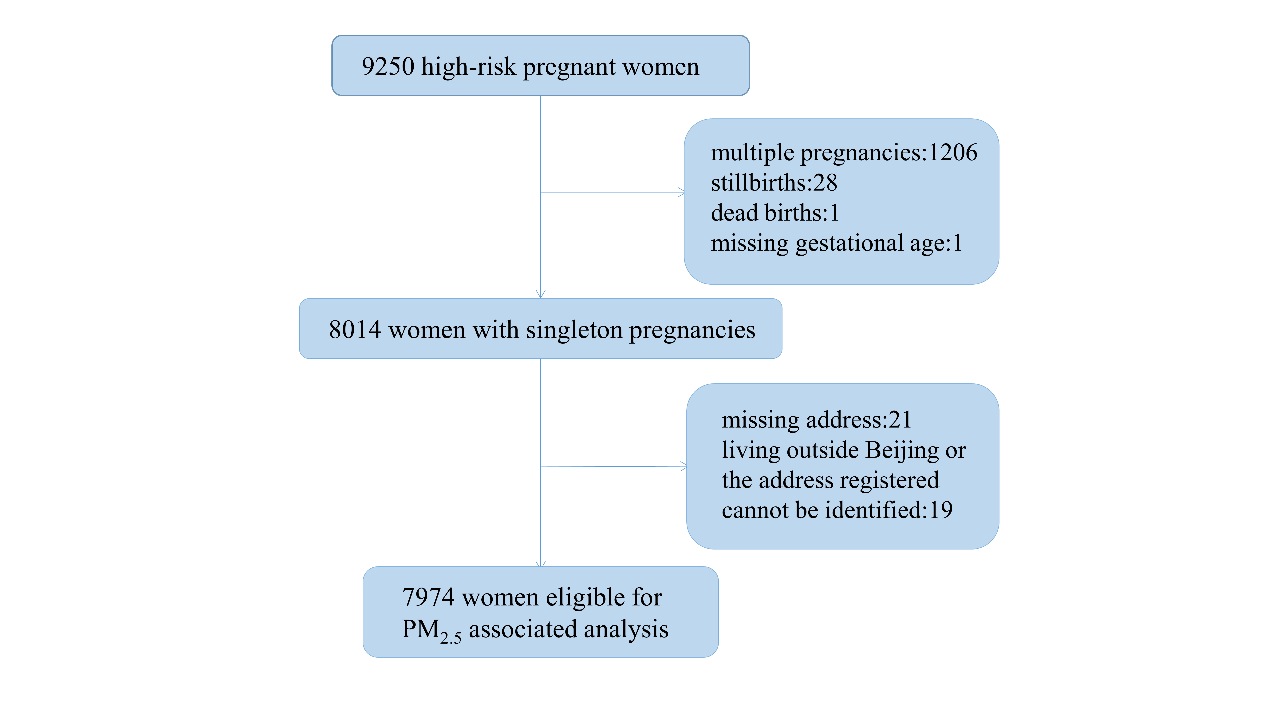


Fig. S1. Flow chart of study population.

**
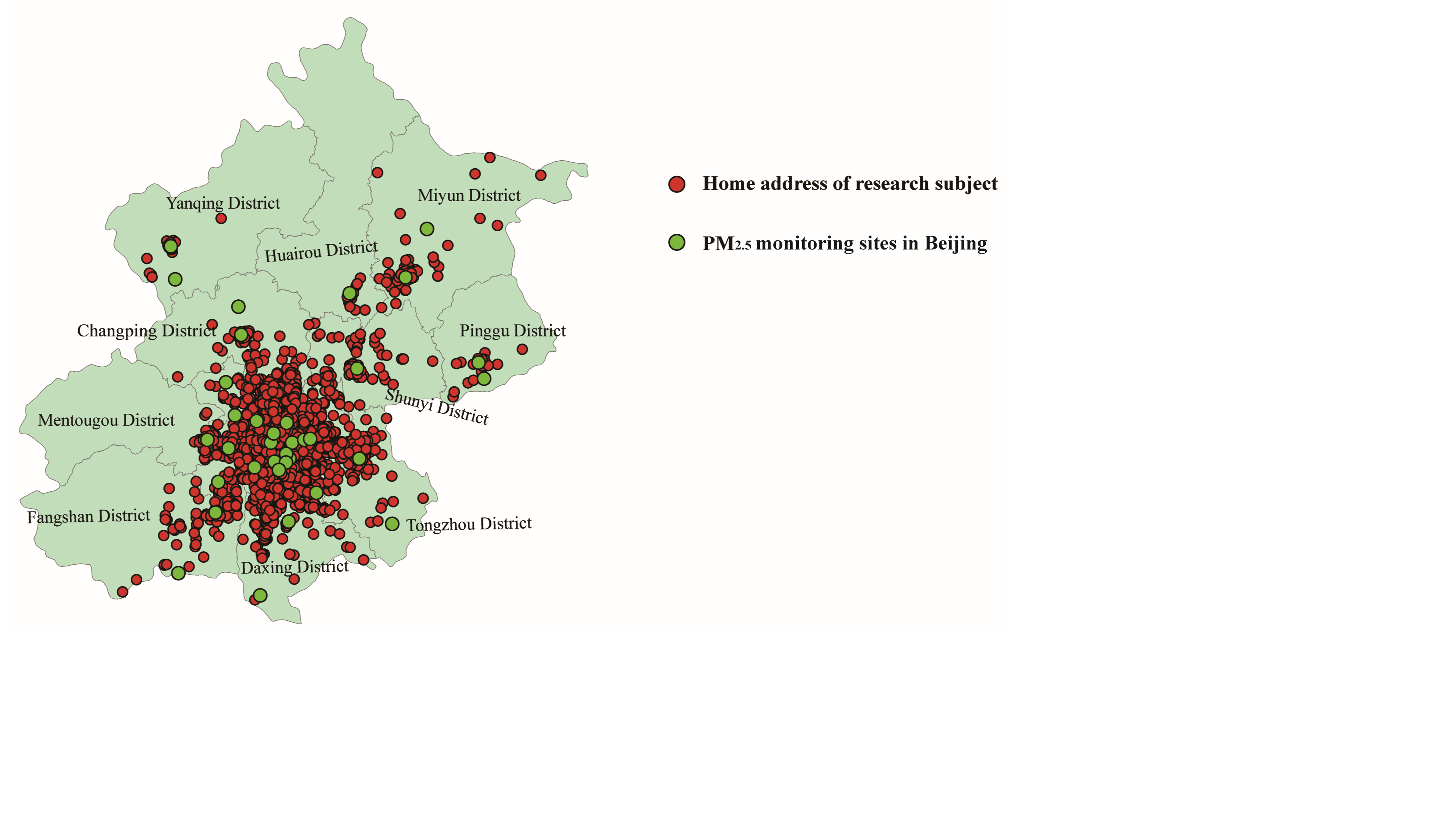
**

Fig. S2. The geographical distribution map of the research objects’ home addresses and nearby monitoring sites in Beijing.
